# Sacroiliac Joint Ankylosis Decreases Intervertebral Fusion Rate in L5/S1 Single Intervertebral Transforaminal Lumbar Interbody Fusion

**DOI:** 10.7759/cureus.39455

**Published:** 2023-05-24

**Authors:** Masaki Tatsumura, Tomoki Koide, Yosuke Ogata, Hiroki Ito, Katsuya Nagashima, Yosuke Takeuchi, Fumihiko Eto, Toru Funayama, Masashi Yamazaki

**Affiliations:** 1 Department of Orthopedic Surgery and Sports Medicine, Tsukuba University Hospital Mito Clinical Education and Training Center, Mito Kyodo General Hospital, Mito, JPN; 2 Department of Orthopedic Surgery, Institute of Medicine, University of Tsukuba, Tsukuba, JPN

**Keywords:** stress concentration, mechanical stress, posterior lumbar interbody fusion (plif), fusion failure, l5/s1, single segment, union rate, transforaminal lumbar interbody fusion (tlif), sacroiliac joint ankylosis

## Abstract

Background

Transforaminal lumbar interbody fusion (TLIF) is a common surgical procedure for lumbar spondylolisthesis and intervertebral foraminal stenosis. Sacroiliac joint ankylosis is also known to occur in patients without axial spondyloarthritis. When sacroiliac joint bony ankylosis occurs and sacroiliac joint mobility is lost, stresses from the lower extremities to the lumbar spine are no longer buffered and are expected to be concentrated between the fifth lumbar (L5) and the first sacral (S1) vertebrae. We hypothesized that sacroiliac joint bony ankylosis could adversely affect L5/S1 intervertebral fusion and investigated the postoperative intervertebral fusion rate in single intervertebral TLIF on L5/S1 among patients with bony ankylosis of the sacroiliac joint.

Methods

Seventy-two patients who had undergone TLIF in the L5/S1 single intervertebral segment since 2014 and had a follow-up of at least one year after surgery were included in the study. Seventy-two patients were divided into the following two groups for comparison: group A consisted of 17 patients with bony ankylosis of the sacroiliac joint on either side on preoperative CT, and group N consisted of 55 patients without ankylosis. We investigated the intervertebral segment fusion rate one year postoperatively. Fisher's exact tests were used for statistical analysis, with a significance level of *P* < 0.05.

Results

Twelve patients (71%) in group A and 50 patients (91%) in group N had a fusion of the L5/S1 intervertebral segment one year after TLIF surgery, with a significantly lower rate in group A (*P *= 0.049).

Conclusions

We conclude that the presence of preoperative sacroiliac joint bony ankylosis is a risk factor for postoperative intervertebral fusion failure after single-segment TLIF at L5/S1.

## Introduction

Transforaminal lumbar interbody fusion (TLIF) is a common procedure for lumbar spondylolisthesis and intervertebral foraminal stenosis. Sacroiliac joint bony ankylosis is also known to occur as a result of degenerative changes without axial spondyloarthritis [1]. A simple radiographic evaluation of the general population shows an incidence of 10.5%, with a higher prevalence in males [2]. It is also known to occur in asymptomatic patients with a prevalence of degeneration of the sacroiliac joint reported to be 65.1% [3]. CT evaluation has shown that the condition increases with age [4] and is more common in the elderly [5].

Type 3 of the Eno classification is defined as an ankylosis of the sacroiliac joint [3]. When sacroiliac joint ankylosis occurs and sacroiliac joint mobility is lost, stresses from the lower extremities to the lumbar spine are no longer buffered, and stress concentrations to the segment between the fifth lumbar (L5) and the first sacral (S1) vertebrae, which is the most caudal intervertebral space of the spine, are expected to occur. We hypothesized that this stress concentration may adversely affect postoperative intervertebral fusion when TLIF is performed at L5/S1. To validate our hypothesis, we investigated the postoperative intervertebral fusion rate in single intervertebral TLIF on L5/S1 among patients with bony ankylosis of the sacroiliac joint.

## Materials and methods

Seventy-two consecutive patients who underwent TLIF in the L5/S1 single intervertebral space for seven years from April 2014 to March 2021 at a single institution and were available for at least one year for postoperative follow-up were included. A well-trained spine surgeon was the primary surgeon or supervising assistant in all surgeries. The causative illnesses that led to surgery were 30 cases of isthmic spondylolisthesis, seven cases of degenerative spondylolisthesis, 33 cases of intervertebral foraminal stenosis, and two cases of canal stenosis recurrence after decompression surgery. Seventy-two patients were divided into the following two groups for comparison. Seventeen patients with bony ankylosis of the sacroiliac joint on either side were classified as group A. Fifty-five patients with no bony ankylosis on either side were classified as group N. Table 1 shows the distribution of the number of patients in the two groups for isthmic spondylolisthesis, degenerative spondylolisthesis, intervertebral foraminal stenosis, and canal stenosis recurrence. 

**Table 1:** Patient characteristics. The distribution of the number of patients in the two groups for isthmic spondylolisthesis, degenerative spondylolisthesis, intervertebral foraminal stenosis, and canal stenosis recurrence.

	Group A	Group N	Total
Isthmic spondylolisthesis	7	23	30
Degenerative spondylolisthesis	1	6	7
Intervertebral foraminal stenosis	9	24	33
Canal stenosis recurrence	0	2	2
Total	17	55	72

TLIF was performed by inserting bilateral pedicle screws into the L5 and S1 vertebrae. Two of polyether ether ketone cages with crushed lamina were used as free autogenous bone between vertebral bodies after bilateral facetectomy. No autologous bone grafting was performed in the posterior lateral space. The S1 screw was inserted bicortically so that the anterior part was positioned at the sacral vertebral promontorium.

The study parameters were age, sex ratio, operative time, intraoperative blood loss, and fusion rate of the L5/S1 intervertebral body one year after TLIF. In addition, for group A, whether bony ankylosis was right-sided, left-sided, or bilateral was determined. The number of cases with a cleft between the cage and endplate, vertebral endplate cysts, and radiolucency around the pedicle screws was also examined to evaluate fusion failure.

The presence or absence of bony ankylosis of the sacroiliac joint was determined by preoperative CT, and only Eno classification type 3 was considered ankylotic, excluding type 1 and type 2 [3]. The anterior, middle, and posterior Gahleitner patterns were all considered ankylotic [5]. Bone fusion between the vertebral bodies after TLIF was determined using CT sagittal and coronal images taken one year postoperatively, as in previous studies [6]. Bone fusion was defined as the presence of bony bridging between the grafted bone and the vertebral body endplate. Bone fusion failure was defined as the absence of bony bridging between the graft and the endplate and the presence of one of the following signs of union failure: cleft between the cage and endplate or vertebral endplate cysts or radiolucency around the pedicle screws [7,8].

Comparisons were made between groups A and N. The Student’s t-test was used to compare age between groups and Fisher’s exact tests were used to compare sex ratio, fusion rate, cleft between the cage and endplate, vertebral endplate cysts, and radiolucency around the pedicle screws. The significance levels for both were set at *P* < 0.05. The retrospective review of medical records and images received the approval of our institutional review board (IRB, no. 22-56).

## Results

The mean age was 72.1 years in group A and 67.2 years in group N (*P *= 0.09). The male-to-female patient ratio was 13:4 in group A and 41:14 in group N (*P *= 0.76). The mean operation time was 215 minutes for group A and 237 minutes for group N (*P *= 0.16). Mean intraoperative blood loss was 400 g in group A and 365 g in group N (*P *= 0.67). Twelve patients in group A (70.6%) and 50 patients in group N (90.9%) had intervertebral bony fusion one year after TLIF surgery, with group A having a significantly lower fusion rate (*P *= 0.049; Table 2). 

**Table 2:** Fusion rate of group A and group N. Postoperative intervertebral fusion rates were 70.6% in group A and 90.9% in group N, with group A having a significantly lower fusion rate (*P *= 0.049).

	Group A	Group N	*P*-value
Mean age (years)	72.1	67.2	0.09
Sex ratio (male:female)	13:4	41:14	0.76
Mean operation time (minutes)	215	237	0.16
Mean blood loss (g)	400	365	0.67
Fusion rate (%)	70.6	90.9	0.049

There were five cases of bone fusion failure in each of the A and N groups. Of these, one case (20%) in group A and four cases (80%) in group N showed a cleft between the cage and endplate (*P *= 0.21); four cases (80%) in group A and five cases (100%) in group N showed vertebral endplate cysts (*P *= 1); and two cases (40%) in group A and three cases (60%) in group N showed radiolucency around the pedicle screws (*P *= 1; Table 3). There were two cases in group A and five cases in group N with overlapping fusion failure signs.

**Table 3:** Fusion failure signs in group A and group N. One case (20%) in group A and four cases (80%) in group N showed a cleft between the cage and the endplate (*P *= 0.21); four cases (80%) in group A and five cases (100%) in group N showed vertebral endplate cysts (*P *= 1); and two cases (40%) in group A and three cases (60%) in group N showed radiolucency around the pedicle screws (*P *= 1).

	Group A	Group N	*P*-value
Cleft between the cage and the endplate	1	4	0.21
Vertebral endplate cysts	4	5	1.0
Radiolucency around the pedicle screws	2	3	1.0

There were six cases of lateralization of bony ankylosis in group A on the right side, five cases on the left side, and six cases bilaterally.

Complications such as surgical site infection and implant failure did not occur in either group.

Representative cases

Case 1

**Figure 1:**
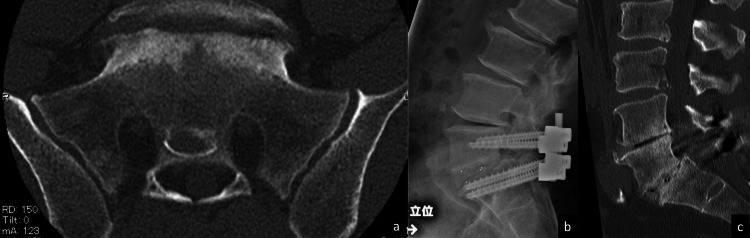
Case 1: A case without bony ankylosis of the sacroiliac joint. Bone fusion was achieved one year after surgery: (a) preoperative axial CT slice showed the absence of bony ankylosis; (b) postoperative X-ray during extension showed no sign of bone fusion failure around the endplates; and (c) postoperative sagittal CT showed bone continuity between L5 and S1 endplates.

A 62-year-old man with a chief complaint of right lower extremity pain was diagnosed with isthmic spondylolisthesis of the fifth lumbar vertebra. He had no bony ankylosis of the sacroiliac joint before surgery, and TLIF of the L5/S1 was performed. One year after surgery, bony fusion was confirmed by X-ray and CT (Figure 1).

Case 2

**Figure 2:**
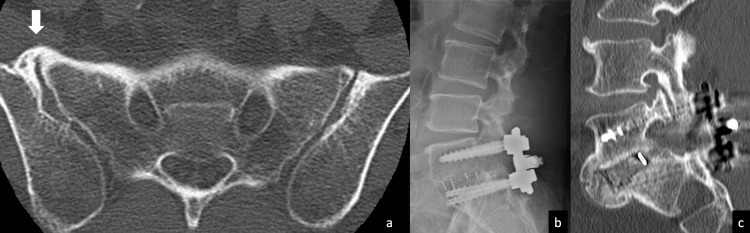
Case 2: A case with anterior bony ankylosis of the right sacroiliac joint. Bone fusion was achieved one year after surgery: (a) preoperative axial CT slice showing bony ankylosis of the right sacroiliac joint. The white arrow indicates the bony bridge. (b) Postoperative X-ray during extension showing no sign of bone fusion failure around the endplates. (c) Postoperative sagittal CT showing a bone bridge between the L5 and S1 endplates.

A 59-year-old man with a chief complaint of left lower extremity pain was diagnosed with isthmic spondylolisthesis of the fifth lumbar vertebra. Before surgery, anterior bony ankylosis of the right sacroiliac joint was observed. One year after TLIF of L5/S1 was performed, bony fusion was confirmed by X-ray and CT (Figure 2).

Case 3

**Figure 3:**
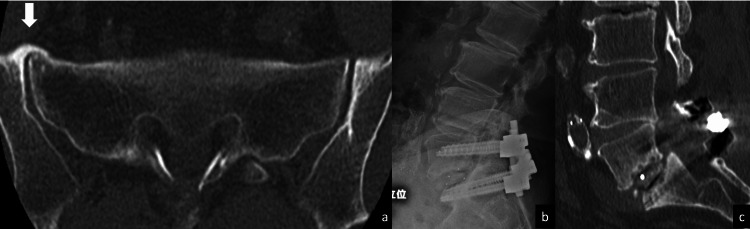
Case 3: A case with anterior bony ankylosis of the right sacroiliac joint. Bone fusion was not achieved even one year after surgery: (a) Preoperative axial CT slice showing bony ankylosis of the right sacroiliac joint. The white arrow indicates the bony bridge. (b) Postoperative X-ray during extension showing the cleft around the cages. (c) Postoperative sagittal CT showing the cleft above the endplates and vertebral endplate cysts.

A 72-year-old man with a chief complaint of left lower extremity pain was diagnosed with isthmic spondylolisthesis of the fifth lumbar vertebra. Before surgery, anterior bony ankylosis of the right sacroiliac joint was observed; TLIF of L5/S1 was performed, and even one year after surgery, bony fusion had not occurred based on X-ray and CT images (Figure 3).

## Discussion

While sacroiliac joint ankylosis is a known cause of low back pain, many cases are asymptomatic [3]. In this study, latent sacroiliac joint ankylosis was found in 23.6% of the TLIF cases at the L5/S1 single vertebral segment. While a relatively large number of patients are affected, there have not been many previous investigations. There was a nonsignificant trend toward older age in the ankylosis group in this study. As sacroiliac joint ankylosis is reported to increase with age [4] and to be more common in the elderly [5], we hypothesize that ankylosis is due to the aging of surgical patients.

Sacroiliac joint ankylosis is known to result from degenerative changes [3] and tends to be associated with spontaneous spinal intervertebral fusion [9]. Adult patients with sagittal imbalance have a high incidence of sacroiliac joint ankylosis [10]. As spinal imbalance and reduced flexibility also make the spine more susceptible to spinal disease, ankylosis of the sacroiliac joint may predispose to having spinal disease. It has been reported that bony ankylosis anterior to the sacroiliac joint tends to be associated with spinal disease because the mechanical stress on the anterior part of the sacroiliac joint is increased in patients with poor posture due to spinal degeneration [5]. In this study, 42% of the patients had spondylolisthesis. It is estimated that spondylolisthesis occurs in 43% to 74% of cases of isthmic spondylolysis pseudarthrosis [11]. These factors suggest that spinal surgery for patients with sacroiliac joint ankylosis is common and cases in which TLIF is indicated after aging will be increased.

Based on the present results, the intervertebral fusion rate of TLIF was low at L5/S1 in the group with sacroiliac joint ankylosis. The loss of sacroiliac joint mobility results in a stress concentration between the L5/S1 vertebrae due to unattenuated stresses from the lower extremities to the lumbar spine; a link between sacroiliac joint degeneration and degeneration of the L4/5 and L5/S1 intervertebral discs has been reported [4], suggesting that mechanical load distribution may be involved. We hypothesize that this stress concentration disturbs postoperative intervertebral fusion in TLIF for L5/S1.

There was no difference between the two groups in fusion failure signs such as a cleft between the cage and endplate, vertebral endplate cysts, and radiolucency around the pedicle screws.

Pseudarthrosis is one of the most common complications of lumbar spine surgery. Pseudoarthrosis after intervertebral fusion can cause back pain or neurological symptoms, which may lead to revision surgery [12]. In this study, latent sacroiliac joint ankylosis was present in more than 20% of the subjects. Based on the results of this study, we believe that the presence of preoperative sacroiliac joint bony ankylosis is one of the risk factors for postoperative pseudoarthrosis between vertebrae when L5/S1 single intervertebral segment TLIF is performed.

A limitation of this study is its small sample size at a single institute. We were able to investigate only 72 patients who underwent TLIF in a single segment between L5 and S1 during 2014-2021. Since TLIF for L5/S1 is common, a multicenter study should be conducted to investigate a larger sample. Although sagittal plane alignment may play a role in clinical outcomes, alignment was not measured in this study. Additionally, as the follow-up is only one year in this study, we believe that long-term postoperative observation, such as five years, would further clarify the trend.

## Conclusions

The fusion rate of TLIF on the L5/S1 one year after surgery was 70.6% in the ankylosis group and 90.9% in the non-ankylosis group, with a significantly lower rate in the ankylosis group. Pseudoarthrosis after intervertebral fusion can cause back pain or neurological symptoms, which may lead to revision surgery. We conclude that the presence of preoperative sacroiliac joint bony ankylosis is a risk factor for postoperative intervertebral fusion failure after TLIF in a single segment at L5/S1.
